# Back pain and musculoskeletal pain as public health problems: Rural communities await solution

**DOI:** 10.7189/jogh-11-01007

**Published:** 2021-11-06

**Authors:** Anand A Bang, Shekhar Y Bhojraj, Abhay T Bang

**Affiliations:** 1 Center for Spine and Musculoskeletal Care, SEARCH, Gadchiroli, India; 2 Spine Foundation, Mumbai, India

Three decades ago, the people of Gadchiroli, a tribal & rural district from Central India voiced in the annual Health Assembly that pain in the back and extremities (PBE) was one of their most important health priorities. Today, this resonates well with the evidence that back and musculoskeletal pain are world over the leading causes of long term suffering, disability, loss of productivity and expenditure on medical care including cause of visits to health care providers [1-7]. As heavy physical labour is a known risk factor for PBE, the agrarian communities are at higher risk of PBE [8]. Surprisingly, though more than two-thirds of India’s population is rural, there was a dearth of systematic population based research on the burden of PBE. This *Series* of seven community-based studies from two villages in the Gadchiroli district provide for the first time the multidimensional evidence on the prevalence, epidemiology, clinical patterns, health care seeking, disability and economic losses due to PBE in a rural community.

These studies reveal an extraordinarily high prevalence of PBE with nearly five out of six adults suffering from PBE during the preceding 12 months. The economic loss, including due to work-days lost, medical consultation and purchasing medications equal to 4.9% of the annual income. Pain was predominantly of chronic nature (mean 166 painful days in a year) and at multiple sites (mean 4.6 sites per adult) with female sex, agrarian occupation and increasing age were the key risk factors. This means as the life expectancy of India increases, the burden of PBE may increase. Interestingly, non-specific back and neck pain, soft tissue rheumatism (STR) and arthritis were the commonest clinical diagnoses, while specific infectious and inflammatory disorders as well as serious spinal disorders such as myelopathy were rare. Though PBE predominantly resulted in mild to moderate disability, agrarian tasks for both men (60%) and women (64%) and household tasks for women (69%) were significantly associated with moderate to severe disability. Primary care seeking was from private / informally trained practitioners (64.6%) as well as application of home remedies (61.6%) whereas care seeking from the public health facilities was considerably less (17%). Overall, these studies clearly highlight the devastating implications of pain with resultant disability and economic loss for a population for which the primary source of livelihood is manual labour.

These community based intensive studies complement the recent Global Burden of Diseases 2017 [9] estimates wherein pain in back, neck and osteoarthritis have been identified as major causes of years lived with disability.

What could be the possible next steps? Undoubtedly, there is need of more population based studies from different parts of rural India to identify the regional estimates of and the epidemiological variation in the profile of PBE. Ergonomic and clinical studies and measuring the clinical or subclinical nutritional deficiencies are needed for the identification of risk factors to understand the causation pathway for PBE. Similarly, as these studies have demonstrated for the first time the need and use of an indigenously developed questionnaire to measure disability due to back pain in rural communities, local and culture specific scales should be developed for all the joints.

But in parallel, these studies underline the urgent need to prioritize pain relief as the public health priority. Delivery of optimal pain alleviation services through the public health system is necessary as an essential component of primary care. Accessibility to analgesia cannot wait for the majority of the rural Indians. Hence the development of evidence based and culturally appropriate interventions which respond to the expectations of patients should be an immediate research priority. Such intervention models may need to be multidisciplinary, including increasing the availability of generic analgesics, access to physiotherapy in community, ergonomic advice, as well as development and propagation of devices using appropriate technology to promote behaviour change. Counselling to help patients cope better with pain and address the co-existing mental distress, with which the pain may have a bi-directional association, may be crucial.

A key finding from these studies is that though surgical intervention is needed for a select minority of patients, most of the cases of PBE are non-surgical and in fact the pain is non-specific, which is considered as Nociplastic Pain as per a recent plan classification [10,11]. Such pain may remain lifelong, and without a specific biomarker. Therefore, such pain can and in fact must be managed through community based care models, involving trained community health workers, with specialists required only for the select complex cases or those involving co-morbidity. Such task shifting is feasible as demonstrated especially in maternal, newborn and child health care, and in fact essential, considering the huge burden of PBE. Hence the development of appropriate algorithms which allow syndromic diagnosis of pain as well as identification of the specific red flags to triage the patients from the community will be needed.

What should be the outcomes of such interventions? Not necessarily complete pain relief. Instead, the focus should be on improving functionality and reducing disability, ensuring “return-to-work”, reducing economic cost including work-days lost and medical expenditure, and improving the overall quality of life [12].

There will be significant challenges in delivering the interventions. First and foremost is the sheer scale of the problem to be addressed. Creating multidisciplinary teams to develop individualized plan on a mass scale will severely challenge the available resources. A community which is constantly engaged in manual labour may not comprehend the importance and difference with physiotherapeutic exercises. Ensuring the compliance of treatment, especially of physiotherapy and to reduce the reliance on medications would be challenging. Addressing the individual’s expectation of complete relief will surely test the health care provider.

Nevertheless, as these studies unequivocally point out, prevention of these disorders, health education of adult population and analgesia is a fundamental need and the decentralization and delivery of pain management cannot wait further.
